# The Treatment of Whooping Cough

**Published:** 1891-07-04

**Authors:** 


### THE PRACTITIONER'S MIRROR AND RETROSPECT.

[*The Editor will be glad to receive offers of co-operation and contributions from members of the profession. All letters should be addressed to* THE EDITOR, THE LODGE, PORCHESTER SQUARE, LONDON, W.]

### MEDICINE.

### THE TREATMENT OF WHOOPING COUGH.

The various methods of treatment for this very common and, in one sense, most fatal complaint are well worth consideration and comparison. They are numerous, they are very diverse, many of them nevertheless do really show results of great value, but in many of them we fear that special fallacies due to the nature of the problem have led to all sorts of benefits being attributed to perfectly useless remedies. Indeed, the therapeutic art existing in any country or possessed by an individual may be estimated very fairly by the usual treatment employed for whooping cough.

We have here an infective febrile complaint depending on a specific germ, and running a regular course which is apt to be attended with serious and fatal complications. Now in the way of prevention and disinfection there are serious difficulties, otherwise it would be our rational course to thus anticipate the enemy rather than to wait his attack. The contagium is present at such an early stage, it is so powerful, it is so easily carried in the atmosphere and in bundles of clothes or preserved in fomites that very little can be done in the way of isolation. The glorious hope that means may be found for rendering us immune or protected beforehand against specific disease germs is only fulfilled in a few cases, and, as yet neither the germ of measles, nor any weakened vaccine have been discovered. We are, therefore, cut off from the best of all our methods, that of prevention. We can neither stamp out the disease, nor protect against it. As in so many other complaints, we have to take a lower position and fight the enemy when he has thrown himself into the citadel of the body.

We may either seek for a specific remedy, which in very few diseases is discoverable, while our knowledge of the causes is imperfect; or we may simply aid nature by putting the patient in the most favourable conditions for resisting; or we may place him under favourable conditions and then utilise remedies which have a known therapeutic value for complications. The danger is lest we are drawn into a mere treatment of symptoms where these are unimportant instead of attacking the causes, and again of mistaking the benign course of the disease for the result of some useless remedy we have employed. Now, in measles the last is a most frequent fallacy. For its course is so irregular, and improvement comes so suddenly that every sort of nostrum is put forward with confidence as a means of cutting short its course. Statistics and the opinions of men of great experience are accumulated proving that every fresh remedy is superior to all the rest, till we wonder how a single germ of the disease is left alive. When we find that tons of poppy petals are sold as oil of kermes and vouched for as efficacious, and that two-thirds of the drugs in the pharmacopœia are declared on good evidence to be real remedies, we begin, with Kingsley, to despair of ever getting rid of the lies out of history. The empirical search for a specific remedy while we know nothing of the cause of the disease, and again the difficulty of distinguishing the effect of drugs in such a variable malady are at the root of much of this waste of time.

Sometime ago it was stated that children who were placed among the exhalations of gas lime were cured immediately, and endless remedies in the form of vapours of carbolic and salcylic acids were employed with more or less apparent success.

But the evidence is inconclusive; even the gas lime chamber seems uncertain, and the general drift of therapeutic search has since tended elsewhere. The next great class of agents are the nerve sedatives. Belladonna, bromides, and such powerful drugs as antimony and aconite are tested with the limited aim of stopping or lessening the paroxysms, yet Fagge remarks that the ordinary ones cannot be depended on to produce any effect in all, or even in almost all, cases. We spend five or six days in getting the patient under the full influence of belladonna or carbolic acid, and if any improvement occurs, we cannot tell with certainty whether it is due to the drug or no. It is clear that till some more certain or rational method is arrived at, till we can isolate Letzerich's or some other germ, or can demonstrate the pathology on which the symptoms depend, it is desirable to choose the least dangerous of these additions to general hygienic treatment.

Antipyrin has recently been lauded as a fairly reliable sedative. Genser found in 120 cases that the average duration with antipyrin treatment was 24 days, instead of from 60 to 90, and this is confirmed by others, but it does not seem any more exempt from danger than belladonna or carbolic acid. Phenacetin and antifebrin have a similar power in controlling the paroxysms, and in equivalent doses are, perhaps, neither more nor less safe. Bromoform produced by the action of bromine on alcohol in the presence of an alkali, and brought forward as an anæsthetic, has been recently employed and recommended by Stepp, of Nuremberg, and Lowënthal, of Berlin. They report that the vomiting ceased and the course of the disease was shortened. Three to four drops were given three times daily to children from two to four years of age in a teaspoon of water, and when used at an early stage the improvement was very rapid and marked. However, it seems to have the ordinary risks of chloroform in producing coma, and a fall of blood pressure accompanied by deep inspirations when given in toxic doses. There is, besides, the additional risk of bromine being set free if the mixture is exposed to light. If, too, according to a well known law, the activity of allied elements increases with their atomic weight bromoform is a more powerful drug than chloroform, as well as much less known in its effects. A remarkable pl[illegible]n of treatment was recommended a short time ago by a German authority, which has the merit of perfect harmlessness. During a paroxysm, the hyoid bone with the tongue is pressed up and forwards by the thumbs placed below the jaw. The paroxysm is said to be rendered abortive and the little patient soon recognises the relief given and begs for its repetition. Anything which reduces the number and violence of the coughs without counterbalancing evils reduces the strain on the system. Probably, belladonna in full and increasing doses is as safe as any drug of this class, but we incline, where possible to trust to simple astringents for the irritated nerve endings. Where a case can only be seen at intervals, such remedies, with perhaps some bromide and an expectorant in view of bronchial dangers, is probably all we can attempt, but where the patient is under frequent observation we may be called upon to try the stronger sedatives if the paroxysms are violent. The exhibition of vapour of terebene or pine oil is at once a safe and frequently successful adjunct to general treatment. If the weather is fine there is good reason to think that better results are obtained by out of door life, but the greatest care must be taken to avoid chills.

In reviewing the various proposed drugs, if we are struck with the uncertainty of the results, we must conclude that a surer way to success will be the discovery of the true pathology of the disease, and in place of these unsatisfactory trials of sedatives, we may then fix precisely the object of our remedy.

